# Brain Activation during Thoughts of One’s Own Death and Its Linear and Curvilinear Correlations with Fear of Death in Elderly Individuals: An fMRI Study

**DOI:** 10.1093/texcom/tgab003

**Published:** 2021-01-28

**Authors:** Kanan Hirano, Kentaro Oba, Toshiki Saito, Shohei Yamazaki, Ryuta Kawashima, Motoaki Sugiura

**Affiliations:** 1 Institute of Development, Aging and Cancer, Tohoku University, Sendai 980-8575, Japan; 2 International Research Institute of Disaster Science, Tohoku University, Sendai 980-8572, Japan

**Keywords:** death anxiety, functional MRI, mortality, older adults, self

## Abstract

Facing one’s own death and managing the fear of death are important existential issues, particularly in older populations. Although recent functional magnetic resonance imaging (fMRI) studies have investigated brain responses to death-related stimuli, none has examined whether this brain activation was specific to one’s own death or how it was related to dispositional fear of death. In this study, during fMRI, 34 elderly participants (aged, 60–72 years) were presented with either death-related or death-unrelated negative words and asked to evaluate the relevance of these words to the “self” or the “other.” The results showed that only the left supplementary motor area (SMA) was selectively activated during self-relevant judgments of death-related words. Regression analyses of the effect of fear of death on brain activation during death-related thoughts identified a significant negative linear correlation in the right supramarginal gyrus (SMG) and an inverted-U-shaped correlation in the posterior cingulate cortex (PCC) only during self-relevant judgments. Our results suggest potential involvement of the SMA in the existential aspect of thoughts of death. The distinct fear-of-death-dependent responses in the SMG and PCC may reflect fear-associated distancing of the physical self and the processing of death-related thoughts as a self-relevant future agenda, respectively.

## Introduction

Facing one’s own death and managing the fear of one’s own death are important existential, psychological, and clinical issues, particularly for older people. According to the French philosopher Jankélévitch (1966), the concept of death can be regarded from 3 different perspectives: death in the first person (my own death), death in the second person (your death, the death of someone close or of a loved one), and death in the third person (the other’s death, death in general). One’s own death is unique from an existential point of view because it is associated with “the most extreme possibility,” that is, that one could no longer exist in any shape or form and will thus be unable to be involved with or engage in anything that exists in this world (Heidegger 1996). Considerations of one’s own death are crucial because this process involves such existential issues.

The cognitive processes underlying the existential aspect of thoughts of death (i.e., those thoughts relevant to one’s own nonexistence) have attracted much attention in the past few decades. In psychology, terror management theory (TMT; Greenberg et al. 1986) postulates the involvement of defense mechanisms, referred to as proximal defenses, during thoughts of death. For example, when people consciously contemplate their own death, they suppress death-related thoughts or deny their vulnerability and attempt to remove such thoughts from focal attention (Pyszczynski et al. 1999). In neuroscience, recent functional magnetic resonance imaging (fMRI) studies have addressed brain responses related to the existential aspect of thoughts of death and found increased or decreased activity in several brain regions. For example, increased activity in response to death-related statements or words (vs. negative stimuli) was observed in the right amygdala (Quirin et al. 2012), the dorsal anterior cingulate cortex (ACC)/supplementary motor area (SMA; Klackl et al. 2018), and the left inferior frontal cortex (Qin et al. 2018). Increased activity in the right ventrolateral prefrontal cortex was observed in a mortality salience (vs. negative affect) treatment group during a word-relationship task (Yanagisawa et al. 2016). In contrast, decreased activity was reported in the bilateral insular cortices for death-related (vs. negative) stimuli or tasks (Han et al. 2010; Shi and Han 2013; Klackl et al. 2014; Qin et al. 2018). Both increase (Quirin et al. 2012) and decrease (Qin et al. 2018) in the activity of the ACC have been observed.

However, these fMRI studies did not dissociate brain responses related to the existential aspect of thoughts of death from other aspects of thoughts of death, such as death in the second person or death in the third person, because they did not perform manipulation of the self-relevance of the thoughts. This may be because a psychological perspective like TMT and most of the abovementioned fMRI studies have assumed that both self- and other-death thoughts share the same existential process. However, considering the philosophical perspective of Jankélévitch (1966) who differentiates self- and other-death, it is possible to assume an existential process only on self-death and a separate process on other-death thoughts. Regarding other-death, it is assumed that although death in the second person may have a comparable impact with death in the first person, “I” is still alive. Conversely, death in the third person may be general, abstract, and virtually anonymous; it may be relevant to serenity rather than to tragedy. Death is also considered a taboo subject for many people in the modern society (Gorer 1965; Sabri and Obermiller 2012), including in Japan (Hirayama 1991). Besides, the own-death-specific process is believed to be distinct from that of such general death, based on the difference in event-related potentials between self-death and other’s death (Fan and Han 2018).

It is considered reasonable to focus on elderly individuals when seeking to reveal the neural bases of the existential aspect of thoughts of death. This population is temporally closest to death, and therefore the realization of one’s own death might be stronger. Many psychological studies have investigated the attitudes of older adults toward death (e.g., Wong et al. 1994; Cicirelli 2002; Kawashima 2005) and their perspectives or preferences regarding end-of-life issues (e.g., Catt et al. 2005; Malcomson and Bisbee 2009; Ke et al. 2017). The psychosocial developmental theory (e.g., Erikson 1963, 1982) suggests that a main developmental task for elderly individuals is to cope with their own mortality without fear.

Indeed, this line of research would be more meaningful when focusing on the individual differences in dispositional fear (or anxiety, avoidance) of death, which in turn may influence one’s quality of life or dying process in various ways. For example, anxiety or fear of death among older individuals is associated with psychological problems (Gilliland and Templer 1986; White and Handal 1991; Wong et al. 1994; Thorson and Powell 2000). Additionally, fear or avoidance of thinking/talking about death has been associated with less effective end-of-life preparations among healthy elderly people (Carr and Khodyakov 2007; Glass and Nahapetyan 2008). Furthermore, fear of death is a target of intervention during palliative care (National Consensus Project for Quality Palliative Care 2018).

There exist distinct hypotheses on the cognitive processes associated with fear of death during thoughts of one’s own death. First, a positive linear relationship may be expected between emotional responses and fear of death. In fact, a positive correlation between activation and death anxiety was reported in the insula (Shi and Han 2013), an emotion-related brain area; however, the self-death-specificity of this relationship was not examined. Second, a negative linear relationship may be expected between processing of self-body representation and fear of death based on psychological studies that have suggested fear of death is associated with body awareness (e.g., Arndt et al. 1998; Bourdin et al. 2017). Third, non-linear (inverted-U-shaped) relationships may be expected between thinking of the future and fear of death. As with a high level of death anxiety (Carr and Khodyakov 2007; Glass and Nahapetyan 2008), a low level of death anxiety could be maladaptive because it can also reflect avoidance or denial of impending death in patients with a life-threatening illness (Furer and Walker 2008; Tong et al. 2016; Curran et al. 2017). Conversely, adaptability in those with a moderate level of death anxiety could be explained by their tendency to engage in planning for the future (Tong et al. 2016).

In sum, the present study used fMRI to identify brain responses while one is thinking about his/her own death (i.e., existential aspect of thoughts of death) and individual differences in brain responses associated with dispositional fear of death during thoughts of one’s own death. We recruited elderly individuals, as we consider them the most appropriate target population for this particular subject. The neural bases of the existential aspect of thoughts of death were examined using a self-relevant judgment task (Craik et al. 1999; Kelley et al. 2002). In this experiment, participants were asked to make both self- and other-relevant judgments of death-related words and death-unrelated negative words. Comparisons between self-related and other-related processes within the context of death-related thoughts allowed for the identification of self-specific thoughts of death. Regarding fear-of-death-related brain activity, the linear and non-linear (U-shaped or inverted-U-shaped) relationships between fear of death and self-death-specific brain activity were assessed. The degree of fear of death in each participant was measured using a questionnaire. In addition to self-death-specific activity, the common activation of self and other death-related responses was examined to confirm whether self-death thoughts share the same process with other-death thoughts.

It was hypothesized that brain region(s) that is(are) associated with self-death-specific activity and the common activation of self and other death-related activity would be observed in a different subset of brain regions previously implicated in cognitive processes underlying thoughts of death, such as the amygdala, ACC, SMA, inferior frontal cortex, and insular cortex (Han et al. 2010; Quirin et al. 2012; Shi and Han 2013; Klackl et al. 2014, 2018; Yanagisawa et al. 2016; Qin et al. 2018). Regarding the positive linear correlation between brain activation and fear of death, activation in emotion-related region(s), such as the insula (Craig 2009), would be expected. Regarding negative linear correlations between brain activation and fear of death, involvement of region(s) implicated in self-body representation, such as the right supramarginal gyrus (SMG; e.g., Di Vita et al. 2016), would be expected. Regarding the inverted-U-shaped correlations between brain activation and fear of death, region(s) that are considered involved in self-relevant future thinking such as the medial prefrontal cortex and/or posterior cingulate cortex (PCC; Johnson et al. 2006; Addis et al. 2007) would be expected to be activated.

## Materials and Methods

### Participants

This study analyzed data from 34 older adults (range: 60–72 years old, mean [*M*] = 66.3, standard deviation [*S.D.*] = 3.9; 19 men and 15 women) who were recruited by posting an announcement in a local town paper. All participants were right-handed native Japanese speakers, had a high school-level or higher education, were not using medications for high blood pressure or diabetes, and had no past or present history of neurological or psychiatric illness. Additionally, each participant scored a 27 or higher on the Mini-Mental State Examination (Folstein et al. 1975; Sugishita 2012) and a 4 or lower on the Geriatric Depression Scale (Sheikh and Yesavage 1986; Matsubayashi and Ozawa 1994). The details regarding the screening tests and exclusion criteria can be found in the Supplementary Methods.

The study was approved by the Institutional Review Board of the Graduate School of Medicine of Tohoku University, Japan, and was conducted in accordance with the Declaration of Helsinki. Written informed consent was obtained from all participants.

### Questionnaires

Prior to participating in the fMRI experiment, all participants completed the 7-item Fear of Death subscale, which measures negative thoughts and feelings regarding death (e.g., “I have an intense fear of death”), in the death attitude profile-revised (DAP-R; Wong et al. 1994; Kumabe 2006). The scores may range from 1 to 5, with higher scores indicating a greater fear of death; participants had an average score of 2.1 (*S.D.* = 0.8) and the Cronbach’s alpha coefficient was 0.894. We also subjected the Fear of Death scores to a Shapiro–Wilk test for normality (Shapiro and Wilk 1965) using SPSS Statistics 23.0 (IBM Corp.) and confirmed that, statistically, the scores in the present study followed a normal distribution (*P* = 0.067), which allowed performing regression analyses.

Participants also completed the Cognitive Reappraisal scale and the Expressive Suppression scale of the emotion regulation questionnaire (ERQ; Gross and John 2003; Yoshizu et al. 2013). Because the aim of the present study was to investigate brain responses specific to death-related stimuli, the scores on each subscale were used as covariates of no interest when analyzing the fMRI data to minimize the influence of individual differences in the habitual use of these emotion regulation strategies, regardless of the type of stimuli. See Supplementary Methods for further details regarding the questionnaires.

### Stimuli

The fMRI experiment included 40 death-related words (e.g., sudden death, cremation, and metastasis) and 40 death-unrelated negative words (e.g., stomachache, cold, and eye disease). All words consisted of 2 Japanese Kanji (Chinese characters) in noun form. The 2-word categories were matched for arousal, emotional valence, imageability, familiarity, self-relevance, and response times (RTs) but significantly differed only in terms of death-relatedness, with the mean rating for death-related words being more than twice higher than that for death-unrelated words. See Supplementary Methods for the selection procedure.

### Task and Procedure

A factorial 2 × 2 mixed block/event-related design composed of task type (self-relevant judgment and other-relevant judgment, henceforth referred to as “Self task” and “Other task,” respectively) and stimulus type (death-related words and death-unrelated words) was adopted. As a result, the following 4 conditions were created: 1) SD—self-relevant judgment regarding death-related words; 2) SND—self-relevant judgment regarding death-unrelated words; 3) OD—other-relevant judgment regarding death-related words; and 4) OND—other-relevant judgment regarding death-unrelated words.

**Figure 1:**
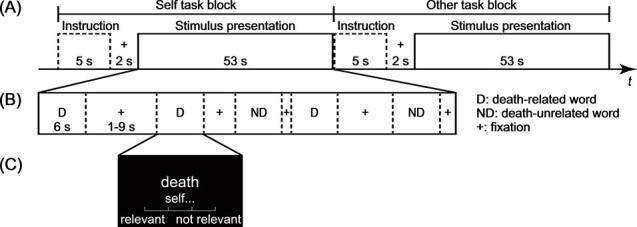
Mixed block/event-related design of the fMRI experiment. (*A*) Timeline of a functional run. Participants alternated between the Self task block and the Other task block during each fMRI run. (*B*) Timeline of a single task block. Within each task block, participants were presented with a total of 5 words (2 or 3 of the 5 words were death-related). Each word was presented for 6 s, during which participants made a judgment about the word. The inter-stimulus interval (ISI) was jittered between 1 and 9 s, during which a fixation cross was presented. (*C*) Example of a single stimulus. Below each stimulus word, a brief instructional reminder was present to indicate the task type and a response reminder (4-point scale); all stimuli and brief reminders were presented in white letters on a black background.

The task design is illustrated in Figure 1. During each run, the participants alternated between the Self task block and the Other task block. In each block, participants were visually presented with death-related words or death-unrelated words for 6 s, during which they made 1 of 2 types of judgments corresponding to the type of block for each word: a self-relevant judgment (“How relevant the word is to yourself”) or an other-relevant judgment (“How relevant the word is to the prime minister”). Participants were encouraged to judge the degree of relevance within the period of the word’s presentation using a 4-point scale (“*relevant*” to “*not relevant*”) by pressing one of 4 response keys with one of their right fingers. The responding fingers were counterbalanced across participants, and responses and RTs were recorded.

The experiment included a total of 4 runs (160 trials in total), each containing 8 task blocks (4 Self and 4 Other task blocks). Each task block lasted for 60 s and began with a 5 s instruction screen that indicated the task the participant was required to complete. The starting order of the task blocks was counterbalanced across participants. Within each task block, 5 words (2 or 3 of the 5 were death-related words) were visually presented. Each stimulus was presented twice during the fMRI experiment (i.e., once in the Self task and once in the Other task), and the word presentation order was pseudo-randomized within each block. The inter-stimulus interval was jittered between 1 and 9 s, during which, a fixation cross was presented. The visual stimulus was projected onto a semi-lucent screen attached to the head-coil of the MRI scanner using PsychoPy 2 (version 1.84.2; Peirce et al. 2019) and was viewed via a mirror.

For self-relevant judgments in the present study, the term “relevant” was defined as “what each word is describing would happen to oneself or is relevant to one’s future,” and the participants were instructed to think of the situation from a future perspective. For the other-relevant judgment, the terms “oneself” and “one’s future” in the definition were replaced with “the prime minister” and “the prime minister’s future,” respectively. In accordance with previous studies (e.g., Craik et al. 1999; Kelley et al. 2002), the “other” was a public figure. In this case, the other was Shinzo Abe, who was the prime minister of Japan at the time of the experiment and was assumed to be well known but not particularly close to the participants; it was expected that the participants would accept his death as a death in the third person perspective. Moreover, the age of the prime minister was within the age range of the participants and was therefore comparable to that of the “self.” Although some studies that have adopted self-relevance judgment tasks used a close other (e.g., a family member or close friend) as a control condition, this option was not selected because it likely would have induced a socioemotional response inherent to death in the second person, rather than death in general, and would have complicated the interpretation of the differences in brain activation.

Following the scanning procedure, behavioral data were acquired to ensure that thoughts of death were more intense for death-related words than for death-unrelated words, and that other emotional or cognitive variables (arousal, emotional valence, and imageability) had not affected self-death-specific activity. See Supplementary Methods for further details regarding the data acquisition process.

Behavioral data were assessed using 2-way repeated-measures analysis of variance with task type (Self task and Other task) and stimulus type (death-related words and death-unrelated words) as factors using SPSS Statistics 23.0 (IBM Corp.).

### fMRI Data Acquisition

During each fMRI session, 38 transaxial images (echo time = 30 ms, flip angle = 81°, slice thickness = 3.0 mm, slice gap = 0.5 mm, field of view [FOV] = 192 mm, matrix = 64 × 64, and voxel size = 3 × 3 × 3 mm) covering the whole cerebrum were acquired with a repetition time of 2500 ms using a gradient-echo echoplanar imaging sequence and a 3-Tesla Philips Achieva scanner (Philips Medical Systems). In each session, 197 volumes were acquired during a total scanning period of 492.5 s. Each participant performed 4 sessions, and in total, 788 volumes were acquired. After all runs, T1-weighted anatomical images (thickness = 1 mm, FOV = 192 mm, and matrix = 240 × 240) were acquired using a magnetization-prepared rapid gradient-echo pulse sequence.

### fMRI Data Analysis

Statistical Parametric Mapping 12 (SPM12; Wellcome Department of Imaging Neuroscience) and MATLAB R2016a (MathWorks) were used for all preprocessing and statistical analyses. The following procedures were performed during preprocessing: correction for head motion, adjustment of acquisition timing across slices, co-registration of the anatomical image to realigned EPI images, segmentation of the structural image into 6 different tissue classes, spatial normalization to the Montreal Neurological Institute template using the co-registered anatomical image and smoothing with an isotropic Gaussian kernel with an 8-mm full-width at half-maximum.

A 2-level approach was adopted for statistical analyses of the event-related fMRI data. In the first-level analysis (fixed effect), the degree of neural activation was estimated based on a voxel-by-voxel multiple regression analysis of the time course. For each participant, a general linear model (GLM) was defined using the hemodynamic response function with the GLM containing regressors that represented the 4 conditions: SD, SND, OD, and OND. Each regressor modeled neural activation during the 6 s period in which the stimulus was presented. Six estimated motion parameters obtained during head movement estimation (3 for translation and 3 for rotation) were also included as covariates of no interest. A high-pass filter with a cutoff frequency of 1/128 Hz was applied for detrending purposes. Although there were a few trials with a missing response for some participants whose data were analyzed, these trials were not considered independent error trials because, considering the low frequency of such trials, it was highly likely that the participants were thinking but failed to respond. Furthermore, using independent models for the error trials would have caused unequal designs across participants because not all participants provided erroneous responses.

Contrast images ([SD–SND]–[OD–OND]) and ([SND–SD]–[OND–OD]) were independently generated for each participant to identify self-specific activation and deactivation for death-related words, respectively (i.e., interactions between stimulus type and task type). To identify common activation and deactivation between self-related activation for death-related words (SD–SND) and other-related activation for death-related words (OD–OND), namely conjunction of (SD–SND) with (OD–OND) and of (SND–SD) with (OND–OD), contrast images SD, SND, OD, and OND were generated for each subject.

In the second-level analysis (random effects), one-sample *t*-tests of the contrast images from each participant were conducted for population inference to identify self-specific activation and deactivation for death-related words. Age, sex, and the scores on the Cognitive Reappraisal and Expressive Suppression (i.e., ERQ scores) were entered as covariates of no interest. The threshold for significant activation was initially set at *P* < 0.001 (uncorrected) and was corrected to *P* < 0.05 for multiple comparisons using cluster size, assuming the whole cerebrum to be the search volume. As a result, significant brain activation was identified for the contrast (SD–SND)–(OD–OND) (i.e., self-specific activation for death-related words). Since the result of the interaction might have been influenced by the effect(s) of other factors, such as deactivation of death-related words in other-relevant judgment tasks, the contrast image ([SD–SND]–[OD–OND]) was inclusively masked by (SD–SND) and (SD–OD) to avoid extracting data on any activation of no-interest and to ensure that the interaction occurred due to the activation being highest specifically in the SD condition. This masking procedure allows for removing of all voxels that do not reach the default level of significance in the masking contrast of (SD–SND) and (SD–OD), respectively, when analyzing the contrast ([SD–SND]–[OD–OND]). In SPM12, the default level of significance for the mask is set to *P* = 0.05, uncorrected. Additionally, conjunction analyses of (SD–SND) and (OD–OND), and of (SND–SD) and (OND–OD) were performed to identify common activation and deactivation between self-related activation for death-related words and other-related activation for death-related words, respectively, where the covariates of no interest and threshold were the same as the one-sample *t*-tests for self-death-specific activation.

To explore the cortical areas in which self-death-specific activity correlated with Fear of Death scores, 2 voxel-wise multiple regression analyses were performed. For the linear and curvilinear (U-shaped or inverted-U-shaped) relationships, scores on the Fear of Death scale were entered as first- and second-order explanatory terms, respectively, with the ([SD–SND]–[OD–OND]) contrast images and age, sex, and scores on the Cognitive Reappraisal and Expressive Suppression subscales being included as covariates of no interest. The statistical threshold was the same as that for the one-sample *t*-tests.

## Results

### Behavioral Results

The behavioral data are summarized in Table 1. As expected, the main effect of stimulus type on thoughts of death was significantly higher for death-related words than for death-unrelated words, which suggests that the manipulation of death-related thoughts was successful. Additionally, the task-by-stimulus interactions were tested to ensure that other emotional or cognitive variables other than thoughts of death did not affect the self-death-specific activity. However, the interactions were significant for some variables other than thoughts of death. Nevertheless, their interaction patterns did not parallel those of activation in the main fMRI results. See Supplementary Results for further details.

**Table 1:** Cognitive or emotional evaluation and RT for each word category per each judgment task in the fMRI experiment

	Self-relevance judgment		Other-relevance judgment		Main effect		Interaction
	Death-related words	Death-unrelated words	Death-related words	Death-unrelated words	Task	Word	
Variable	*M* (*S.D.*)	*M* (*S.D.*)	*M* (*S.D.*)	*M* (*S.D.*)			
Effect of word type							
Thoughts of death	3.70 (1.02)	2.77 (1.14)	3.43 (1.06)	3.01 (1.12)		^**^	^**^^a^
Possible confounding factors							
Arousal	3.50 (1.44)	3.47 (1.27)	3.11 (1.25)	3.41 (0.99)			
Emotional valence	−1.12 (0.68)	−0.54 (0.49)	−0.69 (0.79)	−0.31 (0.39)	^**^	^**^	
Imageability	4.03 (1.02)	4.31 (0.82)	3.07 (1.19)	3.68 (0.89)	^**^	^**^	^*b, c, d^
Relevance	2.46 (0.51)	2.57 (0.49)	2.53 (0.58)	2.74 (0.56)		^**^	
RT (s)	2.59 (0.54)	2.57 (0.49)	2.63 (0.58)	2.53 (0.49)			^*b^

Notes: Means and *S.D.* of cognitive or emotional evaluation and RT are shown in the second to fifth column (left to right). Thoughts of death, arousal, and imageability were rated on a 7-point scale (1 = not at all, 7 = very much). Relevance was rated on a 4-point scale (1 = not relevant, 4 = relevant). Emotional valence was rated on a 7-point bipolar scale (−3 = very negative, +3 = very positive). Relevance ratings and the RTs were obtained during the fMRI scanning, and the remaining variables were obtained after scanning.

^*^ *P* < 0.05; ^**^P < 0.01.

^a^The simple main effect of stimulus type was significant in the Self task.

^b^The simple main effect of stimulus type was significant in the Other task.

^c^The simple main effect of task type was significant in the “death-related words” condition.

^d^The simple main effect of task type was significant in the “death-unrelated words” condition.

### fMRI Results: Self-Death-Specific Activity Changes

**Figure 2:**
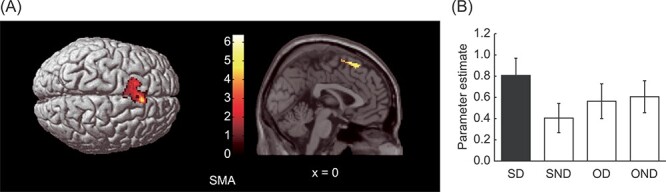
Brain areas showing self-death-specific activity. (*A*) The left SMA shows significant activation in the contrast image ([SD–SND]–[OD–OND]). The left panel shows activation rendered on the top surface of the brain. The right panel shows activation superimposed on a sagittal section of the anatomical image. Color bars represent *t*-values. The threshold was uncorrected *P* < 0.001, corrected to *P* < 0.05 using the cluster size. (*B*) The activation profile represents the parameter estimate under each condition relative to the baseline in the peak voxel in the left SMA (−18, 16, 62). Error bars represent the standard error.

There was significant self-death-specific activation in the left SMA (Table 2 and Fig. 2) but no significant self-death-specific deactivation (Table 2).

**Table 2:** Brain areas showing significant changes in self-death-specific activity

Contrast		Coordinates			Peak-level	Cluster-level	
Hemi	Region	*x*	*y*	*z*	*t*-Value	*k*	*P* (FWE)
(SD–SND)–(OD–OND) masked by (SD–SND) and (SD–OD)							
L	Supplementary motor area	−18	16	62	6.33	332	0.009
		0	16	62	5.46		
		2	26	56	5.26		
(SND–SD)–(OND–OD)							
	No suprathreshold voxels						

Notes: Coordinates (*x*, *y*, *z*) of the activation peak in the Montreal Neurological Institute space, peak *t*-value, size of the activated cluster in number (*k*) of voxels (2 × 2 × 2 mm), and *P*-value (corrected to *P* < 0.05 using cluster size) at the peak are given.

Hemi = hemisphere, L = left. FWE = family wise error.

### fMRI Results: Common Activity Changes of Self- and Other-Death

**Figure 3:**
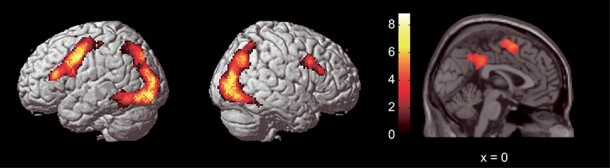
Brain areas showing common activity of self- and other-death. The first 2 panels (left to right) show activation rendered on the lateral surface of the left and right hemispheres, respectively. The third panel shows activation superimposed on the sagittal section of the anatomical image. Color bars represent *t*-values. The threshold was uncorrected *P* < 0.001, corrected to *P* < 0.05 using the cluster size.

Significant increases in activation related to thoughts of death, that is, common activation of (SD–SND) and (OD–OND), were observed in several regions (Table 3 and Fig. 3), including a large activation cluster in the right occipito-temporo-parietal region that included peaks in the right inferior occipital gyrus, superior occipital gyrus, superior parietal lobule, fusiform gyrus, angular gyrus, and PCC. Another large activation cluster was also observed in the left occipito-temporo-parietal region and contained peaks in the left inferior occipital gyrus, superior parietal lobule, fusiform gyrus, and angular gyrus. We also observed a large activation cluster in the left frontal region, which included peaks in the left inferior frontal sulcus and precentral gyrus. Further, activations were observed in the left SMA and right inferior frontal sulcus. Decreases in activation related to thoughts of death, that is, common deactivation of (SD–SND) and (OD–OND), were not observed.

**Table 3:** Brain areas showing significant changes in common activity of self- and other-death

Contrast		Coordinates			Peak-level	Cluster-level	
Hemi	Region	*x*	*y*	*z*	*t*-Value	*k*	*P* (FWE)
Conjunction of (SD–SND) and (OD–OND)							
R	Inferior occipital gyrus	22	−82	−6	8.82	5167	0.000
R	Superior occipital gyrus	32	−78	18	7.67		
R	Fusiform gyrus	42	−42	−20	5.19		
R	Superior parietal lobule	28	−60	44	4.61		
R	Angular gyrus	46	−64	32	4.33		
R	Posterior cingulate cortex	4	−36	38	5.26		
L	Inferior occipital gyrus	−24	−82	−10	8.43	4982	0.000
L	Fusiform gyrus	−38	−40	−20	6.63		
L	Superior parietal lobule	−22	−66	38	5.99		
L	Angular gyrus	−42	−68	30	3.58		
L	Inferior frontal sulcus	−38	8	28	6.82	2418	0.000
L	Precentral gyrus	−42	2	40	6.03		
L	Supplementary motor area	−6	6	56	5.90	805	0.000
R	Inferior frontal sulcus	40	8	30	5.14	521	0.004
Conjunction of (SND–SD) and (OND–OD)							
	No suprathreshold voxels						

Notes: Coordinates (*x*, *y*, *z*) of the activation peak in the Montreal Neurological Institute space, peak *t*-value, size of the activated cluster in number (*k*) of voxels (2 × 2 × 2 mm), and *P*-value (corrected to *P* < 0.05 using cluster size) at the peak are given.

Hemi = hemisphere, R = right, L = left. FWE = family wise error.

### fMRI Results: Effects of Fear of Death on Brain Activity

#### Linear Correlation Analyses

**Figure 4:**
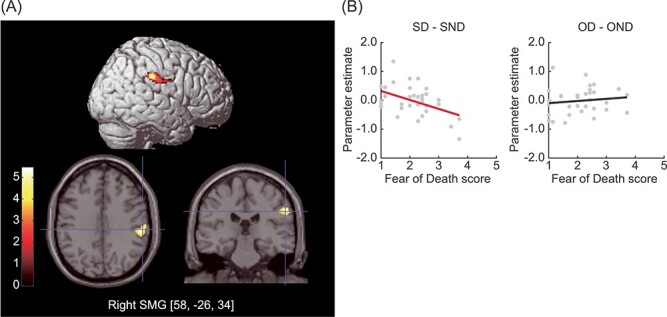
Linear effects of fear of death on self-death-specific activation. (*A*) A significant negative linear correlation between Fear of Death scores and self-death-specific activation ([SD–SND]–[OD–OND]) was observed in the right SMG. The top panel shows activation rendered on the lateral surface of the right hemispheres. The lower 2 panels show activation superimposed on the axial and coronal sections of the anatomical image. Color bars represent *t*-values. The threshold was uncorrected *P* < 0.001, corrected to *P* < 0.05 using the cluster size. (*B*) Parameter estimates under the (SD–SND) and (OD–OND) conditions for the peak voxel in the right SMG (58, −26, 34) were extracted and plotted against the Fear of Death scores. The slope for the SD condition is shown in red.

The correlation analysis revealed a significant negative linear correlation between the scores on the Fear of Death scale and activation in the right SMG in the contrast images ([SD–SND]–[OD–OND]); (Table 4 and Fig. 4). The plot illustrates that the correlation between the degree of fear of death and death-related activation was specific to the Self task. A positive correlation was not identified.

**Table 4:** Linear effects of fear of death on self-death-specific activation

		Coordinates			Peak-level	Cluster-level	
Hemi	Region	*x*	*y*	*z*	*t*-Value	*k*	*P* (FWE)
Positive linear correlation							
	No suprathreshold voxels						
Negative linear correlation							
R	Supramarginal gyrus	58	−26	34	5.42	226	0.039

Notes: Contrast: (SD–SND)–(OD–OND); that is, self-specific activation for death-related words.

Coordinates (*x*, *y*, *z*) of the activation peak in the Montreal Neurological Institute space, peak *t*-value, size of the activated cluster in number (*k*) of voxels (2 × 2 × 2 mm), and *P*-value (corrected to *P* < 0.05 using cluster size) at the peak are given.

Hemi = hemisphere, R = right, FWE = family wise error.

#### Curvilinear Correlation Analyses

**Figure 5:**
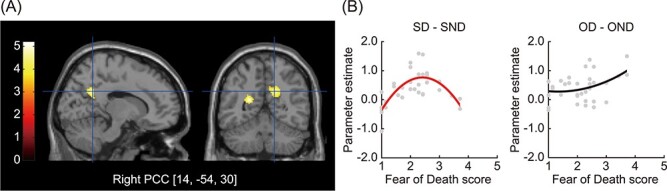
Curvilinear effects of fear of death on self-death-specific activation. (*A*) The right PCC showed a significant inverted-U-shaped correlation between Fear of Death scores and self-death-specific activation ([SD–SND]–[OD–OND]). The left and right panels show activation superimposed on the sagittal and coronal sections of the anatomical image. Color bars represent *t*-values. The threshold was uncorrected *P* < 0.001, corrected to *P* < 0.05 using the cluster size. (*B*) Parameter estimates under the (SD–SND) and (OD–OND) conditions for the peak voxel in the right PCC (14, −54, 30) were extracted and plotted against the Fear of Death scores. Only the fitted curve for the SD condition is shown in red.

The second-order model analysis revealed a significant inverted-U-shaped correlation between the scores on the Fear of Death scale and activation in the right PCC and a cluster that extended from the left PCC to the adjacent ventricle in the contrast images ([SD–SND]–[OD–OND]); (Table 5 and Fig. 5). The plot illustrates that the inverted-U-shaped correlation between the degree of fear of death and death-related activation was specific to the Self task. A U-shaped correlation was not identified.

**Table 5:** Curvilinear effects of fear of death on self-death-specific activation

		Coordinates			Peak-level	Cluster-level	
Hemi	Region	*x*	*y*	*z*	*t*-Value	*k*	*P* (FWE)
U-shaped correlation							
	No suprathreshold voxels						
Inverted-U-shaped correlation							
R	Posterior cingulate cortex	14	−54	30	4.72	225	0.040
L	Posterior cingulate cortex/Ventricle	−22	−52	16	5.17	245	0.030

Notes: Contrast: (SD–SND)–(OD–OND); that is, self-specific activation for death-related words.

Coordinates (*x*, *y*, *z*) of the activation peak in the Montreal Neurological Institute space, peak *t*-value, size of the activated cluster in number (*k*) of voxels (2 × 2 × 2 mm), and *P*-value (corrected to *P* < 0.05 using cluster size) at the peak are given.

Hemi = hemisphere, R = right, L = left. FWE = family wise error.

## Discussion

The primary aim of this study was to identify brain responses in older adults while they were thinking of their own death (i.e., existential aspect of thoughts of death) and the associations of these brain responses with the dispositional fear of death. Results revealed increases in self-death-specific activity in the SMA. Although a death-related response in this region has previously been reported (Klackl et al. 2018), the present study was the first to show its self-specific nature. Additionally, significant negative linear and inverted-U-shaped relationships with fear of death were self-death-specifically observed in the right SMG and PCC, respectively. Relationships between these regions and fear of death have not been previously reported.

### Self-Death-Specific Activation

The present results provide support for the notion that the SMA/ACC are involved in behavioral inhibition of thoughts of death, as previously suggested (Klackl et al. 2018). This interpretation is based on the notion that the existential threat of mortality highlights mismatches between current or anticipated circumstances and existential needs, and such a mismatch activates a behavioral inhibition system involving the SMA/ACC. Although existential threats should be specific to one’s own death, the previous study did not assess the self-specificity of death-related brain responses. Our results confirmed this interpretation by showing that the response observed in this region was self-death-specific.

However, the conclusion that activation of the SMA reflected behavioral inhibition should be made with caution, as the correlation between SMA activation and Fear of Death scores was not significant (*r* = −0.055, *P* = 0.756). If SMA activation was associated with inhibition derived from a fear of death, there should have been a significant correlation.

A possible alternative interpretation is that activation of the SMA might reflect the essential nature of one’s own death, that is, the extinction of one’s whole being and, therefore, the impossibility of direct engagement or involvement with the world (Heidegger 1996). “I” die (i.e., death in the first person) refers to the fact that one would no longer be able to receive any type of input from the world (ranging from perceptions of the outer world to feedback as a consequence of one’s own actions) or to approach the world, even if one is willing to do so. When an individual is imagining their own death, they may simulate agency error or intention-feedback incongruency in terms of lack of feedback for any intended action in the world. Our observation on the activation of SMA in the present study is in line with previous studies that suggest SMA to play a role in attention to intention of action (Lau et al. 2004), agency error (Yomogida et al. 2010), and intention-feedback incongruency (Sperduti et al. 2011).

### Common Activity Changes of Self- and Other-Death

The present results showed that self-death thoughts share the same process with other-death thoughts. Additionally, activation in the bilateral frontal cortices, as reported in previous studies (Yanagisawa et al. 2016; Klackl et al. 2018; Qin et al. 2018), appeared as the common activation of self- and other-death thoughts in the present study. However, the results should not be interpreted readily as evidence that the activated areas underpin the existential aspect of thought of death, since a similar activation pattern has been reported in previous studies that have investigated the processing of taboo words (Severens et al. 2012; Hansen et al. 2019). We believe that while self-death-specific activity might reflect the essential nature that is logically inferred from thoughts of one’s own death, activation that were found following conjunction analysis might reflect the inhibitory/emotional response to thoughts of death.

### Effects of Fear of Death on Brain Activity: Linear Correlation Analyses

As expected, a negative linear correlation between fear of death and death-related brain activity was self-specifically observed in the right SMG. The involvement of the right SMG in bodily self-representation has often been reported; for example, this region is associated with non-action-oriented body representations (Di Vita et al. 2016), visual imagery of the body (Costantini et al. 2011), and self-face recognition in the mirror (Sugiura et al. 2015). Our finding suggests the presence of neural support for the relationship between bodily self-awareness and fear of death, as previously reported by several psychological studies; for example, individuals in a cubicle with a large mirror spent less time writing about their own death (Arndt et al. 1998), implying that awareness of the bodily aspect of the self provoked defensive reactions. Additionally, a study targeted in community-dwelling elderly adults revealed that a relationship between death anxiety and positive body image exists (Bodner and Bergman 2019). Besides, a virtual reality study found that alterations in body awareness, such as a simulated out-of-body experience, reduced the fear of death (Bourdin et al. 2017). Although this is one possible interpretation of our finding, the negative correlation between fear of death and activation in the right SMG observed in the present study may, therefore, imply that individuals with a strong fear of death may attempt to distance themselves from their physical self when reminded of their own death.

### Effects of Fear of Death on Brain Activity: Curvilinear Correlation Analyses

Consistent with the present hypothesis, this study revealed an inverted-U-shaped correlation between fear of death and brain activity in the PCC during thoughts of one’s own death. The PCC is often implicated in self-relevant thinking of the future (e.g., Addis et al. 2007), even in older adults (Viard et al. 2011). In particular, the PCC exhibited increased activity when participants were thinking of a motivationally self-relevant agenda (Johnson et al. 2006), which also held true in older adults (Mitchell et al. 2009). Our finding may imply that, when reminded of their own mortality, individuals with a moderate fear of death may think of their own death in terms of a self-relevant future agenda, whereas those at either extreme of fear of death (i.e., high or low levels) may not. A qualitative study conducted by Tong et al. (2016) classified patients with advanced or metastatic cancer into 3 groups based on their death anxiety scores (low, moderate, and high) and investigated features of their fear of death. Patients with moderate death anxiety recognized the imminence of death and this influenced their treatment decisions and future plans; however, patients with high or low anxiety were less likely to engage in this type of thinking about the future.

### Implications

The present study has theoretical and practical implications. In terms of its theoretical implications, this was the first study to introduce the first-person perspective on death into fMRI studies of thanatology by dissociating self-death-specific activation from non-self-specific death-related activation, allowing for empirical discussions of psychological responses to reminders of one’s own death and fear of death from an existential point of view. Further, the linear and inverted-U-shaped correlation between fear of death and self-death-specific activation is theoretically significant in that it facilitates a deeper examination of the relationship between fear of death and adaptability. In terms of its practical implications, the present study provides neurocognitive evidence that supports ideas proposed by psychological studies. For example, the findings demonstrating an inverted-U-shaped correlation may support the suggestion that treatments for fear of death need to be matched to the degree of fear of death and that individuals with low fear of death may require special attention because, in some cases, this could reflect avoidance of thinking about one’s own death (Tong et al. 2016).

### Limitations and Future Directions

This study had several limitations. We used simple word stimuli related to death and asked participants to make judgments based on relevance, which raises important issues regarding ecological validity. Additionally, we presumed that thinking of one’s own death entails reflection on one’s own nonexistence (i.e., existential aspect), but this was not accompanied by empirical support. We admit that these issues are critical limitations common across experimental studies in this field when addressing existential issues. Additionally, it remains unclear whether the observed results were specific to older adults, because the present study did not compare the findings in older and younger adults, since its primary aim was to identify self-death-specific neural activity. Further, our results on the activated regions presented in this study should be interpreted cautiously. Although the activation of the SMA might reflect agency error or intention-feedback incongruency, such an interpretation relies on reverse inference and should be treated with caution (Poldrack, 2006); it is possible that the activation might reflect other cognitive processes. While the negative linear correlation of the right SMG with dispositional fear of death might reflect bodily self-representation, we did not assess bodily self- awareness directly, which should be assessed in future studies. Last but not least, we based the interpretation of the inverted-U-shaped correlation between the scores on the Fear of Death scale and activation in the right PCC on the study by Tong et al. (2016). Our conclusion should be approached cautiously bearing in mind that the fear of death scales used in the present study (i.e., DAP-R) and that of Tong et al. (2016) (i.e., DADDS) differ in content, although the basic idea for both questionnaires is the issues that arise from the possibility of one’s nonexistence. Nonetheless, DAP-R measures negative thoughts and feeling about death (general fear about death including fear about after death), whereas DADDS measures fears about the dying process, concerns associated with the loss of time and opportunity, and impact on loved ones. It should also be taken into account that the target population of the present study was community-dwelling healthy elderly, while Tong et al. (2016) targeted patients with advanced cancer.

## Supplementary Material

Supplementary_final_version_tgaa003

## Notes

The authors gratefully acknowledge and wish to thank the study participants who generously offered their time. *Conflict of Interest*: There are no conflicts of interest to declare.
