# Supplementary material for: Brain Activation during Thoughts of One’s Own Death and Its Linear and Curvilinear Correlations with Fear of Death in Elderly Individuals: An fMRI Study
Source: Cereb Cortex Commun. 2021 Jan 28;2(1):tgab003. doi: 10.1093/texcom/tgab003 (PMC8152848; doi:10.1093/texcom/tgab003)
Supplement: Supplementary_final_version_tgaa003 [file supplementary_final_version_tgaa003.docx]

**Supplementary Methods**

**Details of screening tests and excluded participants**

*Screening tests*: The Mini-Mental State Examination (MMSE) (Folstein et al. 1975; Sugishita 2012) is the tool most widely used to screen for cognitive impairment in older adults. Lower MMSE scores indicate a greater degree of general cognitive dysfunction. The Geriatric Depression Scale (GDS-15; Sheikh and Yesavage 1986; Matsubayashi and Ozawa 1994) is a 15-item self-report scale that screens for depression in older individuals, with higher scores indicating more severe depression.

*Excluded participants*: Initially, 48 older adults were recruited for this study, but 14 were excluded due to high GDS scores (n = 7), poor performance in the fMRI task, i.e., 5% missed responses (n = 2), misunderstanding the task (n = 1), insufficient understanding of the word stimuli i.e., more than 10% of responses to whether they can read or understand the stimuli were “*not at all understandable*” (n = 1), and image defects (n = 3). No participants were excluded due to their MMSE scores.

**Details of questionnaires**

*Death Attitude Profile-Revised (DAP-R)*: The degree of fear of death was assessed using the DAP-R (Wong et al. 1994), a multidimensional scale that measures attitudes toward death. The Japanese version of the DAP-R (Kumabe 2006) consists of 27 items across four subscales: Fear of Death, Death Avoidance, Approach Acceptance, and Escape Acceptance. Only the scores for the Fear of Death subscale were used in this study. For each item, participants responded on a 5-point scale (1 = “*disagree*,” 5 = “*agree*”), and the scores for the seven items were averaged to calculate the final score for each individual.

*Emotion Regulation Questionnaire (ERQ)*: The ERQ (Gross and John 2003) assesses the habitual use of two common emotion regulation strategies (i.e., cognitive reappraisal and expressive suppression), each of which has a corresponding subscale. Cognitive reappraisal involves the reinterpretation of a potentially emotion-eliciting situation to change the emotional impact. Expressive suppression is a response-focused strategy that involves inhibiting emotion-expressive behaviors. The Cognitive Reappraisal scale consists of six items (e.g., ‘‘I control my emotions by changing the way I think about the situation I’m in’’), and the Expressive Suppression scale consists of four items (e.g., ‘‘I control my emotions by not expressing them’’). The participants completed the Japanese version of the ERQ (Yoshizu et al. 2013), which is based on a 7-point scale (1 = “*strongly disagree*,” 7 = “*strongly agree*”). The scores for all items were averaged within each subscale, with higher scores indicating a more frequent use of the corresponding strategy. The participants had an average score of 4.9 (*s.d.* = 0.7) for Cognitive Reappraisal and 4.2 (*s.d.* = 0.9) for Expressive Suppression, with Cronbach's alpha coefficients of .753 and .738, respectively.

**Selection procedure of stimuli**

The selection procedure for the words was as follows. First, 330 death-related and death-unrelated words were selected from the categories of “natural phenomenon” and “human activity” from two Japanese lexicons (Ikehara et al. 1997; The National Institute for Japanese Language and Linguistics 2004) in which words are classified and arranged according to their meaning. For the death-unrelated category, the words that were considered unrelated to death, but described negative events were selected. Second, the stimuli were narrowed down and validated through a pre-test, in which an independent group of 18 healthy older adults (range: 60–75 years, *M* = 67.6, *s.d.* = 4.8; 10 men and 8 women) were asked to rate each word on a 7-point scale in terms of self-relevance (1 = “*not relevant at all*,” 7 = “*very relevant*”). For this rating procedure, PsychoPy 2 (version 1.84.2; Peirce et al. 2019) was used to measure response times (RTs). The participants also completed paper-based questionnaires with 7-point scales to rate each word in terms of semantic death-relatedness (1 = “*not at all related*,” 7 = “*very related*”), arousal (1 = “*not at all arousing*,” 7 = “*very arousing*”), emotional valence (-3 = “*very negative,*” 3 = “*very positive*”), imageability (1 = “*not at all imageable*,” 7 = “*very imageable*”), and familiarity (1 = “*not at all familiar*,” 7 = “*very familiar*”).

Although 21 older adults were initially recruited for the pre-test, three were excluded because they misunderstood the task. Of the 18 remaining participants, 9 rated the initial 330 words, and the remaining 9 rated 187 words that were selected based on the ratings of the first group. The scores were subjected to paired t-tests, and based on these results, death-related and death-unrelated words were selected so that the words in the two categories differed significantly only in terms of the mean rating for semantic death-relatedness (death-related words had a higher mean rating than death-unrelated words) and so that the difference in the mean rating for semantic death-relatedness between the words in the two categories was as great as possible. The mean rating was more than twice as high for death-related words than for death-unrelated words (*t*[17] = 16.86, *p* < .001, paired t-test; death-related words: *M* [*s.d.*] = 6.12 [0.48], death-unrelated words: *M* [*s.d.*] = 2.77 [0.75]). These two-word categories did not significantly differ in terms of the mean ratings for self-relevance, arousal, emotional valence, imageability, familiarity, or RT (see Table S1).

**Table S1.** Cognitive or emotional evaluation and response time for each word category in the pre-test

|  |  | Death-related words | |  | Death-unrelated words | |  | *t*-value |
| --- | --- | --- | --- | --- | --- | --- | --- | --- |
| Variable |  | *M (s.d.)* | |  | *M (s.d.)* | |  |  |
| Effect of word type |  |  |  |  |  |  |  |  |
| Death-relatedness |  | 6.12 | (0.48) |  | 2.77 | (0.75) |  | 16.86 ** |
| Possible confounding factors | |  |  |  |  |  |  |  |
| Arousal |  | 4.15 | (0.74) |  | 3.82 | (0.79) |  | 1.68 |
| Emotional valence |  | -0.87 | (0.40) |  | -0.82 | (0.31) |  | 0.76 |
| Imageability |  | 4.99 | (0.80) |  | 4.70 | (0.85) |  | 1.85 |
| Familiarity |  | 3.98 | (0.97) |  | 4.21 | (0.87) |  | 1.77 |
| Self-relevance |  | 3.96 | (0.57) |  | 3.88 | (0.57) |  | 0.86 |
| Response time (s) |  | 4.13 | (1.38) |  | 4.00 | (1.24) |  | 1.30 |

Death-relatedness, arousal, imageability, familiarity, and self-relevance were rated on a 7-point scale (1 = not at all, 7 = very much). Emotional valence was rated on a 7-point bipolar scale (-3 = very negative, +3 = very positive). The degrees of freedom (*df*) for *t*-tests were 17 for all variables.

** *p* < .01.

**Acquisition of post-scanning behavioral data**

For the both the Self task and the Other task the participants were instructed to rate each word on 7-point scales in terms of arousal (1 = “*not at all arousing*,” 7 = “*very arousing*”), emotional valence (-3 = “*very negative*,” 3 = “*very positive*”), thoughts of death (1 = “*did not think about it at all*,” 7 = “*thought about it very much*”), and imageability (1 = “*not at all imageable*,” 7 = “*very imageable*”) based on their interpretation and impression for each word. The instruction for rating thoughts of death in the Self task was: “To what extent did each word triggered any thoughts on your own death?” While for the Other task the participants rated their thoughts on death in response to the question: “To what extent did each word triggered any thoughts on the prime minister’s death.” Finally, participants were asked to evaluate how well they could read and understand each word using a 3-point scale (“*understandable*,” “*not quite understandable*,” and “*not at all understandable*”).

**Supplementary Results**

**Behavioral results**

Trials in which the participants did not respond (i.e., error trials) were excluded from the analyses. The analysis of imageability included the data from only 33 participants because the rating scores for one participant were missing due to a procedural glitch.

The primary purpose of analyzing behavioral data was to check whether our manipulation of the stimulus type was successful. As expected, the main effect of the stimulus type on thoughts of death was significantly higher for death-related words than for death-unrelated words [*F*(1, 33) = 8.794, *p* = .006]. The task-by-stimulus interaction was also significant for thoughts of death [*F*(1, 33) = 9.160, *p* = .005], and a further simple main effect analysis revealed that the ratings for thoughts of death were higher for death-related words than for death-unrelated words in the Self task [*F*(1, 33) =15.546, *p* < .001]. This may be due to the effect of self-death-specific thoughts.

The secondary purpose was to exclude any alternative explanation that the self-death-specific activity reflected emotional or cognitive reactions. Specifically, the analysis was conducted to ensure that the self-death-specific activity did not reflect arousal, emotional valence, imageability, relevance, and RT. The analysis revealed that the task-by-stimulus interactions were significant for RTs [*F*(1, 33) = 4.882, *p* = .034], and imageability [*F*(1, 32) = 4.537, *p* = .041]. However, these interaction patterns did not follow those for activation in the main fMRI results. In other words, neither RT nor imageability were longest/highest or shortest/lowest for death-related words under the Self task condition, which implies that these results were unlikely to have affected the fMRI interaction effect.

**References**

Folstein MF, Folstein SE, McHugh PR. 1975. “Mini-mental state”: A practical method for grading the cognitive state of patients for the clinician. J Psychiatr Res. 12(3):189–198. doi:10.1016/0022-3956(75)90026-6.

Gross JJ, John OP. 2003. Individual differences in two emotion regulation processes: Implications for affect, relationships, and well-being. J Pers Soc Psychol. 85(2):348–362. doi:10.1037/0022-3514.85.2.348.

Ikehara S, Miyazaki M, Shirai S, Yokoo A, Nakaiwa H, Ogura K, Ooyama Y, Hayashi Y. 1997. Goi-Taikei — A Japanese Lexicon. Tokyo, Japan: Iwanami Shoten. [in Japanese].

Kumabe C. 2006. Factors influencing contemporary Japanese attitudes regarding life and death. Japanese J Heal Psychol. 19(1):10–24. doi:10.11560/jahp.19.1_10. [in Japanese].

Matsubayashi K, Ozawa T. 1994. Evaluation on emotion of the elderly. Geriatr Med. 32:541–546. [in Japanese].

Peirce J, Gray JR, Simpson S, MacAskill M, Höchenberger R, Sogo H, Kastman E, Lindeløv JK. 2019. PsychoPy2: Experiments in behavior made easy. Behav Res Methods. 51:195–203. doi:10.3758/s13428-018-01193-y.

Sheikh JI, Yesavage JA. 1986. Geriatric Depression Scale (GDS): Recent evidence and development of a shorter version. Clin Gerontol J Aging Ment Heal. 5(1–2):165–173. doi:10.1300/J018v05n01_09.

Sugishita M. 2012. Mini mental state examination—Japanese. Tokyo, Japan: Nihon Bunka Kagakusha Co., Ltd. [in Japanese].

The Natural Institute for Japanese Language. 2004. Word list by semantic principles, revised and enlarged edition. Tokyo, Japan: Dainippon-Tosho. [in Japanese].

Wong PTP, Reker GT, Gesser G. 1994. Death Attitude Profile– Revised: A multidimensional measure of attitudes toward death. In: Neimeyer RA, editor. Death anxiety handbook: Research instrumentation and application. Washington, DC: Taylor & Francis. p. 121–148.

Yoshizu J, Sekiguchi R, Amemiya T. 2013. Development of a Japanese version of Emotion Regulation Questionnaire. Japanese J Res Emot. 20(2):56–62. doi:10.4092/jsre.20.56. [in Japanese].
